# Supplementary material for: Investigating the neurovascular coupling across multiple motor execution and imagery conditions: a whole-brain EEG-informed fMRI analysis
Source: Neuroimage. Author manuscript; Available in PMC 2025 Oct 11. (PMC12515077; doi:10.1016/j.neuroimage.2025.121311)
Supplement: Supplementary Information [file NIHMS2116416-supplement-Supplementary_Information.pdf]

Supplementary Materials

Task Description: Discrete 1-dimensional (1D) Cursor Control Task

Each session of the cursor control task consists of eight runs of Left-Right (LR) tasks, followed by eight runs of Up-Down (UD) tasks. Each run consists of 25 discrete trials, with target positions randomized across trials. Each trial starts with a yellow rectangle (target) showing up on the screen, indicating the intended movement direction. For LR tasks, the target appears on either the left or right side of the screen, while for UD tasks, it appears at the top or bottom. Subjects are instructed to imagine moving their right hand to move the cursor to the right and their left hand to move it to the left. For upward movement, they imagine moving their right foot, while for downward movement, they are instructed to relax. After two seconds, a pink circle (cursor) appears at the center of the screen and begins moving based on EEG signals. The subject has up to 6 seconds to move the cursor toward the target, or the other invisible target on the opposite side of the screen. If the cursor reaches the visible target within this time, the trial is considered as a hit. If the cursor moves in the opposite direction and reaches an invisible target, the trial is counted as a miss. If the cursor ended up somewhere in between the two targets without hitting either side, the trial is marked as aborted. The trial ends either after the cursor hits a target or after six seconds of cursor movement. After the trial ends, there is a one-second post-feedback period during which the target and cursor remain frozen on the screen. This is followed by a one-second inter-trial interval with a blank screen before the next trial begins.

The online EEG processing pipeline includes a surface Laplacian filter on channels of interest, C3, C4, and Cz, using their four surrounding electrodes. An autoregressive spectral estimation model is then used to extract the alpha band power from a 3 Hz bin centered at 12 Hz. The estimated alpha power from selected channels is used as input to a linear classifier. In the LR task, the classifier calculates the power difference between C4 and C3, while in the UD task, it computes the negative Cz power. The classifier output is further normalized to zero mean and unit standard deviation based on the outputs from the past 30 seconds. The sign and magnitude of the normalized output determine the cursor's movement direction and velocity, where positive values move the cursor to the right (LR task) or up (UD task), while negative values move it to the left (LR task) or down (UD task).

To assess subject performance, Percent Valid Correct (PVC) is used as the inclusion/exclusion criterion. PVC is calculated as the number of hit trials divided by the number of hit and miss trials, excluding aborted trials. Subjects with an average PVC of at least 70% in both LR and UD tasks within a single session are selected for further EEG-fMRI experiments.

EEG Preprocessing: Gradient Artifact (GA) and Ballistocardiogram (BCG) Artifact Correction

The GA was removed from each channel by subtracting a template generated from a sliding average of 21 blocks of the artifact (Allen et al., 2000). The cleaned ECG data was subjected to a bandpass filtering of 0.5 and 30 Hz and downsampled to 250 Hz. The BCG artifact was then addressed by semi-automatically identifying the R peaks on the ECG and subtracting a 21-block sliding window average artifact template from the data (Allen et al., 1998; Allen et al., 2000). Bad intervals were identified through visual inspection and eye movements, and any remaining BCG artifacts were removed by applying an extended Infomax Independent Component Analysis (ICA) (Maggioni et al., 2014).

Unimodal Block fMRI Analysis: General Linear Model (GLM)

The BOLD activity at each voxel was modeled as a linear combination of task and movement regressors - the parameters of head rotation (N=3) and translation (N=3) obtained from the fMRI preprocessing steps - which served as regressors of interest and confounders, respectively. Each task regressor, representing the modeled BOLD response for a specific task type, was obtained by the convolution of the onset of the trials and the canonical hemodynamic response function (HRF), downsampled to match the fMRI sampling frequency (fs = 0.5 Hz), and high-pass temporally filtered with a 128 s cut-off. The canonical HRF employed in the analysis was a two-gamma function with 6s delays of response and 16s delays of undershoot (spm_hrf function, http://www.fil.ion.ucl.ac.uk/spm/, version 12). A scan-based model was used to account for the three consecutive scans performed during the acquisition. Therefore, each scan was modeled separately in the GLM assuming consistency of task-related effects among different sessions.

References

Allen, P.J., Josephs, O., Turner, R., 2000. A method for removing imaging artifact from continuous EEG recorded during functional MRI. Neuroimage 12 (2), 230–239. doi:10.1006/nimg.2000.0599.

Allen, P.J., Polizzi, G., Krakow, K., Fish, D.R., Lemieux, L., 1998. Identification of EEG events in the MR scanner: the problem of pulse artifact and a method for its subtraction. Neuroimage 8 (3), 229–239. doi:10.1016/j.jneumeth.2003.10.009.

Maggioni, E., Arrubla, J., Warbrick, T., Dammers, J., Bianchi, A.M., et al., 2014. Removal of pulse artefact from EEG data recorded in MR environment at 3T Setting of ICA parameters for marking artefactual components: application to resting-state data. PLoS One 9 (11), e112147. doi:10.1371/journal.pone.0112147.

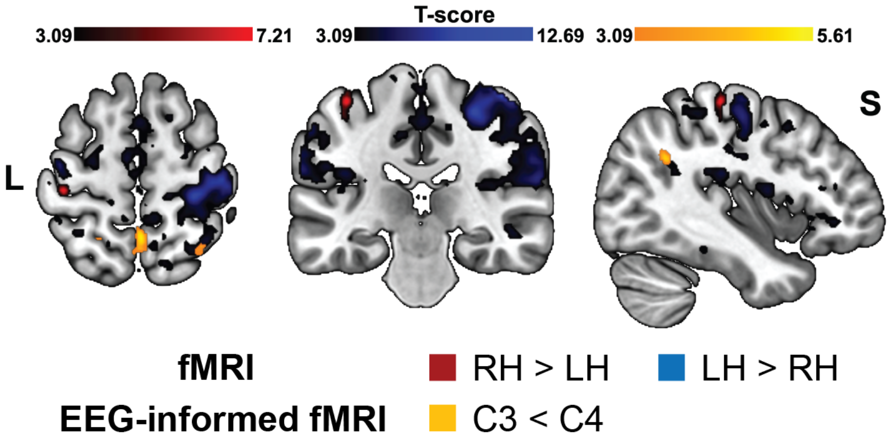

Figure S1. Group-level EEG-informed fMRI statistical maps for ME (C3 < C4; C4 < C3) and MI conditions (C3 < C4) on the entire dataset (n = 17, p<0.001, uncorrected with k higher than the expected number of voxels) [x = -40, y = -26, z = 60]. Results demonstrate robust sensorimotor activation for both ME conditions associated with electrode alpha power. In contrast, MI regressors did not show consistent sensorimotor activity across the full group, instead revealing associated BOLD responses in occipital, temporal, cingulate, and cerebellar regions.

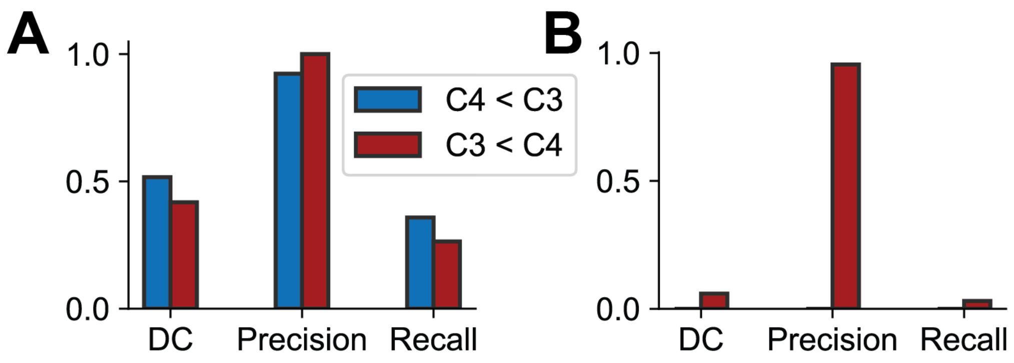

Figure S2. A) Quantitative overlap between fMRI analysis and EEG-informed fMRI analysis results for ME tasks, constrained within the contralateral sensorimotor region (precentral and postcentral gyri), presented as dice coefficient (DC), precision, and recall. **B)** Quantitative overlap between fMRI analysis and EEG-informed fMRI analysis results for MI tasks, constrained within the contralateral sensorimotor region (precentral and postcentral gyri), presented as dice coefficient (DC), precision, and recall.

Table S1. Group averaged ERD values of individual channels for MI tasks (n = 17). Negative values indicate power suppression during the MI tasks, while positive values indicate a power increase during the MI tasks.

Channel	right-hand MI	left-hand MI	Channel	right-hand MI	left-hand MI
Fp1	3.34%	2.33%	P2	3.65%	0.46%
Fp2	2.06%	1.98%	AF3	3.49%	4.40%
F3	4.53%	6.21%	AF4	1.40%	2.88%
F4	-0.87%	-3.98%	FC3	-1.54%	0.21%
C3	-10.33%	-7.72%	FC4	-2.88%	-8.16%
C4	-3.34%	-12.68%	CP3	-9.30%	-4.96%
P3	-3.81%	1.59%	CP4	0.04%	-10.28%
P4	4.03%	-3.90%	PO3	0.71%	5.31%
O1	1.37%	4.35%	PO4	7.60%	2.30%
O2	5.14%	1.05%	F5	6.35%	5.58%
F7	4.71%	1.35%	F6	-4.10%	-1.97%
F8	-2.84%	1.52%	C5	-3.29%	-3.72%
T7	0.64%	-0.84%	C6	-2.11%	-8.29%
T8	-0.61%	-3.23%	P5	-3.64%	1.55%
P7	-1.65%	4.00%	P6	3.11%	-5.08%
P8	1.80%	-3.10%	AF7	5.03%	1.82%
Fz	-1.00%	1.79%	AF8	-0.64%	2.74%
Cz	-1.11%	0.86%	FT7	2.99%	-1.88%
Pz	2.60%	6.90%	FT8	-2.98%	1.87%
Oz	5.73%	3.05%	TP7	-2.00%	1.45%
FC1	-10.70%	-3.64%	TP8	0.91%	-3.95%
FC2	-2.34%	-5.27%	PO7	0.20%	3.46%
CP1	-5.22%	-0.53%	PO8	4.37%	-1.95%
CP2	-0.15%	-5.51%	FT9	2.19%	-0.96%
FC5	2.80%	1.11%	FT10	0.65%	4.22%
FC6	-2.35%	-3.68%	Fpz	2.10%	3.83%
CP5	-6.02%	-3.60%	CPz	-0.79%	3.06%
CP6	-0.17%	-8.24%			
TP9	0.03%	0.82%			
TP10	3.96%	0.17%			
POz	5.25%	9.53%			
F1	5.04%	7.13%			
F2	-1.21%	-0.06%			
C1	-8.60%	-2.83%			
C2	-1.51%	-6.27%			
P1	-1.21%	5.24%			

Table S2. Group-level unimodal fMRI activations for motor execution (ME) using a random effect model with age, sex, and handedness as covariates (p < 0.001, uncorrected with k higher than the expected number of voxels) (n = 17). Labeling performed with AAL3 atlas. p-value associated with the cluster Family-Wise (cFWE) correction is reported in the table.

Contrast	T-value	Cluster-size [# voxels]	Peak coordinates (xyz) [mm]	Atlas regions (%)	p (cFWE)
ME RH > ME LH	10.60	981	-40 -22 60	Left Postcentral gyrus (54), Left Precentral gyrus (44)	<0.001
	7.81	95	-16 -22 4	Left Ventral posterolateral (35), Left Pulvinar lateral (24), Left Intralaminar (14), Left Pulvinar medial (9), Left Mediodorsal lateral parvocellular (8), Left Ventral lateral (6), Left Mediodorsal medial magnocellular (2), Left Pulvinar inferior (1)	ns
	7.74	485	12 -50 -14	Right Lobule IV-V of cerebellar hemisphere (79), Right Lingual gyrus (6), Right Lobule VI of cerebellar hemisphere (5), Lobule IV-V of vermis (5), Right Fusiform gyrus (4)	<0.001
	7.34	87	-32 -10 0	Left Lenticular nucleus-Putamen (89)	<0.05
	6.82	54	-38 -22 22	Left Rolandic operculum (61), Left Insula (39)	ns
	5.58	71	-2 -16 54	Left Supplementary motor area (94), Left Paracentral lobule (6)	ns
	4.81	48	14 -62 -52	Right Lobule VIII of cerebellar hemisphere (88), Right Lobule IX of cerebellar hemisphere (10)	ns
ME LH > ME RH	15.66	770	-12 -48 -16	Left Lobule IV-V of cerebellar hemisphere (73), Left Lobule VI of cerebellar hemisphere (15), Left Fusiform gyrus (7), Lobule IV-V of vermis (3), Left Lingual gyrus (2)	<0.001
	12.12	669	30 -6 0	Right Lenticular nucleus-Putamen (31), Right Rolandic operculum (21), Right Insula (13), Right Lenticular nucleus-Pallidum (6)	<0.001
	11.86	1640	32 -24 64	Right Postcentral gyrus (69), Right Precentral gyrus (24), Right Superior parietal gyrus (3)	<0.001
	8.96	112	8 -10 52	Right Supplementary motor area (100)	<0.05
	8.44	99	16 -22 4	Right Ventral posterolateral (30), Right Pulvinar lateral (28), Right Intralaminar (22), Right Pulvinar medial (8), Right Mediodorsal lateral parvocellular (6), Right Ventral lateral (2), Right Mediodorsal medial magnocellular (2), Right Pulvinar anterior (1)	ns
	6.60	47	-6 -66 -34	Left Lobule VIII of cerebellar hemisphere (72), Lobule VII of vermis (6), Left Crus II of cerebellar hemisphere (4), Lobule VIII of vermis (4)	ns
	6.11	206	-126	Left Lobule VIII of cerebellar hemisphere (95), Left Lobule IX of cerebellar hemisphere (5)	ns
	5.09	12	52 -4 8	Right Rolandic operculum (100)	ns
	5.01	43	6 -22 50	Right Supplementary motor area (93), Right Paracentral lobule (5)	ns
	4.68	28	24 -12 62	Right Superior frontal gyrus-dorsolateral (39), Right Precentral gyrus (32)	ns
	4.35	5	60 0 4	Right Rolandic operculum (100)	ns

Table S3. Group-level unimodal fMRI activations for motor imagination (MI) using a random effect model with age, sex, and handedness as covariates (p < 0.001, uncorrected with k higher than the expected number of voxels) (n = 17). Labeling performed with AAL3 atlas. p-value associated with the cluster Family-Wise (cFWE) correction is reported in the table.

Contrast	T-value	Cluster-size [# voxels]	Peak coordinates (xyz) [mm]	Atlas regions (%)	p (cFWE)
MI RH > MI LH	8.27	19	-2 12 -8	Left Olfactory cortex (63), Left Nucleus accumbens (32), Right Olfactory cortex (5)	ns
	6.35	29	-46 -34 32	Left SupraMarginal gyrus (21), Left Rolandic operculum (3)	ns
	6.22	32	-14 -84 14	Left Cuneus (69), Left Calcarine fissure and surrounding cortex (25), Left Superior occipital gyrus (6)	ns
	6.21	36	-6 -40 32	Left Middle cingulate & paracingulate gyri (92)	ns
	5.75	50	-26 -12 66	Left Precentral gyrus (100)	ns
	5.51	5	-14 -94 20	Left Superior occipital gyrus (100)	ns
	5.44	226	-28 -28 60	Left Postcentral gyrus (60), Left Precentral gyrus (30), Left Superior parietal gyrus (7)	ns
	5.29	18	-32 -4 0	Left Lenticular nucleus-Putamen (94)	ns
	4.99	4	-24 -84 -42	Left Crus II of cerebellar hemisphere (100)	ns
	4.99	6	64 -4 -20	Right Middle temporal gyrus (100)	ns
	4.90	24	-48 -22 46	Left Postcentral gyrus (100)	ns
	4.84	6	4 22 -8	Right Olfactory cortex (100)	ns
	4.83	13	-46 -18 34	Left Postcentral gyrus (77)	ns
	4.80	15	16 -72 8	Right Calcarine fissure and surrounding cortex (100)	ns
	4.76	3	-12 14 -8	Left Nucleus accumbens (100)	ns
	4.70	10	-34 -28 16	Left Rolandic operculum (40), Left Heschls gyrus (40), Left Insula (20)	ns
	4.64	33	24 -64 -16	Right Lobule VI of cerebellar hemisphere (58), Right Fusiform gyrus (21), Right Lingual gyrus (18), Right Lobule IV-V of cerebellar hemisphere (3)	ns
	4.64	4	12 -36 32	Right Middle cingulate & paracingulate gyri (100)	ns
	4.64	8	-40 -22 24	Left Rolandic operculum (88), Left Insula (12)	ns
	4.58	30	14 -48 -18	Right Lobule IV-V of cerebellar hemisphere (97), Right Lobule III of cerebellar hemisphere (3)	ns
	4.55	13	24 -46 -18	Right Lobule IV-V of cerebellar hemisphere (69), Right Fusiform gyrus (31)	ns
	4.52	7	0 -54 -18	Lobule IV-V of vermis (100)	ns
	4.38	10	30 -92 10	Right Middle occipital gyrus (100)	ns
	4.34	8	12 -94 10	Right Calcarine fissure and surrounding cortex (75), Right Cuneus (25)	ns

	4.20	2	4 -74 -38	Right Crus II of cerebellar hemisphere (50), Lobule VIII of vermis (50)	ns
MI LH > MI RH	6.22	59	32 -6 -2	Right Lenticular nucleus-Putamen (93), Right Lenticular nucleus-Pallidum (7)	ns
	5.73	54	30 -22 58	Right Postcentral gyrus (61), Right Precentral gyrus (39)	ns
	5.49	18	42 -26 56	Right Postcentral gyrus (94), Right Precentral gyrus (6)	ns
	4.94	24	38 -14 56	Right Precentral gyrus (100)	ns

Table S4. Group-level unimodal fMRI activations for motor execution (ME) using a fixed effects model (p < 0.001,uncorrected with k higher than the expected number of voxels) (n = 10). Labeling performed with AAL3 atlas. p-value associated with the cluster Family-Wise (cFWE) correction is reported in the table.

Contrast	T-value	Cluster-size [# voxels]	Peak coordinates (xyz) [mm]	Atlas regions (%)	p (cFWE)
ME RH > ME LH	39.58	3229	-38 -24 64	Left Postcentral gyrus (51), Left Precentral gyrus (35), Left Superior parietal gyrus (7), Left Superior frontal gyrus-dorsolateral (2), Left Paracentral lobule (1)	<0.001
	16.93	1218	14 -50 -18	Right Lobule IV-V of cerebellar hemisphere (50), Right Lobule VI of cerebellar hemisphere (16), Lobule IV-V of vermis (11), Right Lingual gyrus (9), Right Fusiform gyrus (7), Lobule VI of vermis (4), Right Lobule III of cerebellar hemisphere (2)	<0.001
	13.08	566	12 -62 -46	Right Lobule VIII of cerebellar hemisphere (80), Right Lobule IX of cerebellar hemisphere (12), Lobule VIII of vermis (6)	<0.001
	8.05	399	-2 -12 56	Left Supplementary motor area (67), Left Paracentral lobule (30), Left Middle cingulate & paracingulate gyri (2)	<0.001
	7.79	148	-14 -22 6	Left Ventral posterolateral (26), Left Pulvinar lateral (19), Left Intralaminar (18), Left Ventral lateral (11), Left Pulvinar medial (11), Left Mediodorsal medial magnocellular (7), Left Mediodorsal lateral parvocellular (7)	<0.001
	7.65	156	-30 -10 0	Left Lenticular nucleus-Putamen (75)	<0.001
	7.07	223	-42 -22 20	Left Rolandic operculum (62), Left Insula (24), Left Postcentral gyrus (9), Left Superior temporal gyrus (5)	<0.001
ME LH > ME RH	39.12	4625	38 -24 66	Right Postcentral gyrus (42), Right Precentral gyrus (25), Right Supplementary motor area (12), Right Superior parietal gyrus (6), Right Superior frontal gyrus-dorsolateral (4), Right Paracentral lobule (2), Right Middle cingulate & paracingulate gyri (1), Right Precuneus (1)	<0.001
	18.25	2112	-16 -50 -20	Left Lobule IV-V of cerebellar hemisphere (38), Left Lobule VIII of cerebellar hemisphere (22), Left Lobule VI of cerebellar hemisphere (17), Left Lingual gyrus (5), Left Fusiform gyrus (5), Left Lobule IX of cerebellar hemisphere (4), Lobule IV-V of vermis (4), Lobule VI of vermis (2)	<0.001
	9.03	989	38 -16 20	Right Rolandic operculum (40), Right Lenticular nucleus-Putamen (23), Right Insula (16), Right SupraMarginal gyrus (2), Right Lenticular nucleus-Pallidum (2), Right Heschls gyrus (1), Right Superior temporal gyrus (1)	<0.001
	6.36	93	16 -20 8	Right Ventral posterolateral (41), Right Pulvinar lateral (25), Right Intralaminar (15), Right Pulvinar medial (10), Right Mediodorsal lateral parvocellular (6), Right Ventral lateral (2), Right Medial Geniculate (1)	<0.05
	4.47	20	4 6 38	Right Middle cingulate & paracingulate gyri (95), Left Middle cingulate & paracingulate gyri (5)	ns
	4.38	30	-52 26 0	Left Inferior frontal gyrus-triangular part (100)	ns
	4.31	143	-40 -54 32	Left Angular gyrus (75), Left Inferior parietal gyrus-excluding supramarginal and angular gyri (7), Left SupraMarginal gyrus (4)	ns
	4.28	29	4 -46 18	Right Posterior cingulate gyrus (83), Right Precuneus (7)	ns
	4.20	17	-66 -40 -6	Left Middle temporal gyrus (100)	ns
	4.00	9	-34 64 0	Left Superior frontal gyrus-dorsolateral (100)	ns
	3.97	15	-64 -22 34	Left SupraMarginal gyrus (93), Left Postcentral gyrus (7)	ns
	3.96	11	-2 64 4	Left Superior frontal gyrus-medial (100)	ns
	3.92	17	-38 34 -10	Left IFG pars orbitalis (100)	ns
	3.74	18	-28 46 -8	Left Superior frontal gyrus-dorsolateral (50), Left Middle frontal gyrus (28)	ns
	3.74	11	-42 -76 -34	Left Crus I of cerebellar hemisphere (82), Left Crus II of cerebellar hemisphere (18)	ns
	3.72	10	-14 -54 30	Left Precuneus (40), Left Middle cingulate & paracingulate gyri (10)	ns

3.67	27	-6 -66 20	Left Precuneus (52), Left Cuneus (48)	ns
3.56	12	58 0 8	Right Rolandic operculum (100)	ns
3.54	23	8 -56 28	Right Precuneus (91), Right Posterior cingulate gyrus (4), Left Precuneus (4)	ns
3.54	6	-12 -56 42	Left Precuneus (100)	ns
3.25	15	-6 -64 36	Left Precuneus (100)	ns

Table S5. Group-level unimodal fMRI activations for motor imagination (MI) using a fixed effects model (p < 0.001,uncorrected with k higher than the expected number of voxels) (n = 10). Labeling performed with AAL3 atlas. p-value associated with the cluster Family-Wise (cFWE) correction is reported in the table.

Contrast	T-value	Cluster-size [# voxels]	Peak coordinates (xyz) [mm]	Atlas regions (%)	p (cFWE)
MI RH > MI LH	17.29	3113	-38 -22 58	Left Postcentral gyrus (50), Left Precentral gyrus (32), Left Superior parietal gyrus (8), Left Paracentral lobule (3), Left Superior frontal gyrus-dorsolateral (2), Left Supplementary motor area (1)	<0.001
	7.80	594	18 -48 -22	Right Lobule IV-V of cerebellar hemisphere (67), Right Lobule VI of cerebellar hemisphere (13), Right Fusiform gyrus (10), Right Lingual gyrus (5), Lobule IV-V of vermis (4), Right Lobule III of cerebellar hemisphere (1)	<0.001
	6.91	242	12 -60 -46	Right Lobule VIII of cerebellar hemisphere (80), Right Lobule IX of cerebellar hemisphere (20)	<0.001
	6.09	144	-30 -6 -2	Left Lenticular nucleus-Putamen (83), Left Insula (1)	<0.001
	4.94	160	-4 0 50	Left Supplementary motor area (81), Left Paracentral lobule (18)	<0.001
	4.88	217	-46 -20 18	Left Rolandic operculum (54), Left SupraMarginal gyrus (19), Left Postcentral gyrus (18), Left Insula (6)	<0.001
	4.36	26	28 68 8	Right Superior frontal gyrus-dorsolateral (92), Right Superior frontal gyrus-medial (8)	ns
	4.26	29	8 68 16	Right Superior frontal gyrus-medial (90), Right Superior frontal gyrus-dorsolateral (7), Left Superior frontal gyrus-medial (3)	ns
	4.21	23	6 8 -8	Right Nucleus accumbens (91)	ns
	4.19	13	-60 -48 38	Left Inferior parietal gyrus-excluding supramarginal and angular gyri (62), Left SupraMarginal gyrus (38)	ns
	4.04	27	-32 -88 -4	Left Middle occipital gyrus (52), Left Inferior occipital gyrus (41)	ns
	4.01	25	54 -62 36	Right Angular gyrus (92), Right Inferior parietal gyrus-excluding supramarginal and angular gyri (8)	ns
	3.98	26	-6 68 14	Left Superior frontal gyrus-medial (81), Left Superior frontal gyrus-dorsolateral (19)	ns
	3.93	120	28 28 44	Right Superior frontal gyrus-dorsolateral (62), Right Middle frontal gyrus (38)	ns
	3.91	18	-26 -80 -44	Left Crus II of cerebellar hemisphere (100)	ns
	3.86	28	-58 -44 30	Left SupraMarginal gyrus (100)	ns
	3.83	24	6 58 -14	Right Superior frontal gyrus-medial orbital (100)	ns
	3.77	33	14 -78 6	Right Calcarine fissure and surrounding cortex (100)	ns
	3.75	15	0 -40 28	Left Posterior cingulate gyrus (73), Left Middle cingulate & paracingulate gyri (20), Right Middle cingulate & paracingulate gyri (7)	ns
	3.74	21	6 -52 -4	Lobule IV-V of vermis (76), Right Lingual gyrus (19), Right Lobule IV-V of cerebellar hemisphere (5)	ns
	3.74	28	-12 -92 -8	Left Calcarine fissure and surrounding cortex (79), Left Lingual gyrus (18), Left Inferior occipital gyrus (4)	ns
	3.73	10	4 20 -8	Right Olfactory cortex (90), Left Anterior cingulate cortex-subgenual (10)	ns
	3.72	6	-26 -82 -20	Left Crus I of cerebellar hemisphere (67), Left Fusiform gyrus (17), Left Lobule VI of cerebellar hemisphere (17)	ns
	3.72	5	10 -84 -38	Right Crus II of cerebellar hemisphere (100)	ns
	3.70	28	4 -64 36	Right Precuneus (89), Left Precuneus (11)	ns
	3.62	39	40 26 44	Right Middle frontal gyrus (100)	ns
	3.58	17	-16 -62 48	Left Superior parietal gyrus (100)	ns
	3.58	9	-4 0 60	Left Supplementary motor area (100)	ns

	3.57	9	22 -72 -20	Right Lobule VI of cerebellar hemisphere (100)	ns
	3.54	6	-16 -24 8	Left Pulvinar lateral (83), Left Pulvinar medial (17)	ns
	3.51	14	-14 -78 -18	Left Lobule VI of cerebellar hemisphere (71), Left Lingual gyrus (21), Left Crus I of cerebellar hemisphere (7)	ns
MI LH > MI RH	18.18	1869	38 -20 58	Right Postcentral gyrus (55), Right Precentral gyrus (36)	<0.001
	6.15	315	-16 -48 -22	Left Lobule IV-V of cerebellar hemisphere (89), Left Fusiform gyrus (7), Left Lingual gyrus (2), Left Lobule VI of cerebellar hemisphere (2)	<0.001
	5.33	214	10 -8 50	Right Supplementary motor area (100)	<0.001
	5.28	87	-28 -50 -54	Left Lobule VIII of cerebellar hemisphere (100)	<0.05
	4.24	40	44 -18 20	Right Rolandic operculum (100)	ns
	4.13	55	30 -6 0	Right Lenticular nucleus-Putamen (100)	ns
	3.47	14	58 -4 8	Right Rolandic operculum (100)	ns
